# CMV2U‐Net: A *U*‐shaped network with edge‐weighted features for detecting and localizing image splicing

**DOI:** 10.1111/1556-4029.70033

**Published:** 2025-04-03

**Authors:** Arslan Akram, Muhammad Arfan Jaffar, Javed Rashid, Salah Mahmoud Boulaaras, Muhammad Faheem

**Affiliations:** ^1^ Faculty of Computer Science and Information Technology The Superior University Lahore Pakistan; ^2^ Information Technology Services University of Okara Okara Pakistan; ^3^ Department of Mathematics, College of Science Qassim University Buraydah Saudi Arabia; ^4^ VTT Technical Research Centre of Finland Espoo Finland

**Keywords:** digital forensics, feature fusion, image authentication, image forgery localization, image splicing detection, *U*‐shaped network

## Abstract

The practice of cutting and pasting portions of one image into another, known as “image splicing,” is commonplace in the field of image manipulation. Image splicing detection using deep learning has been a hot research topic for the past few years. However, there are two problems with the way deep learning is currently implemented: first, it is not good enough for feature fusion, and second, it uses only simple models for feature extraction and encoding, which makes the models vulnerable to overfitting. To tackle these problems, this research proposes CMV2U‐Net, an edge‐weighted U‐shaped network‐based image splicing forgery localization approach. An initial step is the development of a feature extraction module that can process two streams of input images simultaneously, allowing for the simultaneous extraction of semantically connected and semantically agnostic features. One characteristic is that a hierarchical fusion approach has been devised to prevent data loss in shallow features that are either semantically related or semantically irrelevant. This approach implements a channel attention mechanism to monitor manipulation trajectories involving multiple levels. Extensive trials on numerous public datasets prove that CMV2U‐Net provides high AUC and *F*_1_ in localizing tampered regions, outperforming state‐of‐the‐art techniques. Noise, Gaussian blur, and JPEG compression are post‐processing threats that CMV2U‐Net has successfully resisted.

**Highlights**

- A hybrid model combining Canny edge detection with deep learning enhances splicing forgery detection.
- Channel attention improves localization, distinguishing altered regions from the background.
- The model is robust against blurring, compression, and noise, ensuring reliable forensic analysis.
- U‐Net with MobileNetv2 efficiently extracts features, improving performance on forensic datasets.
- Ablation studies confirm structural optimization, outperforming existing methods in forensic tasks.

## 1 INTRODUCTION

Thanks to solid computers, sophisticated picture editing tools, and high‐resolution photographic equipment, changing digital photographs has become relatively easy nowadays. The evolution of image processing technologies, programs, and algorithms has resulted in more false images that are difficult to detect without tools [1, 2]. Tools for altering and fabricating digital photos also grow more accessible as digital images circulate daily. Consequently, techniques that can identify image counterfeiting are much needed. Several approaches have been suggested to achieve this aim [3, 4]. In other approaches, knowledge is ingrained in the picture from the start so that it may be investigated as needed and the legitimacy of the image can be ascertained. Active detection techniques are these. Still, an essential study issue in recent years has been realizing the originality of the image without regard to past embedded knowledge or the main content of the image. These techniques are sometimes referred to as passive or blind ones.

Many phony photos have been generated with the expansion of image processing technologies. Most of the time, these false images are undetectable to the human eye, so image forgery detection techniques are needed. A sound picture forgery detection system should be able to correctly determine the image's authenticity without knowing anything about it or its content. There is a pressing need to detect picture forgeries in many fields, such as scientific research, insurance, medical photography, publication photography, criminal investigations, news agencies, and legal and judicial investigations [5, 6].

**FIGURE 1:**
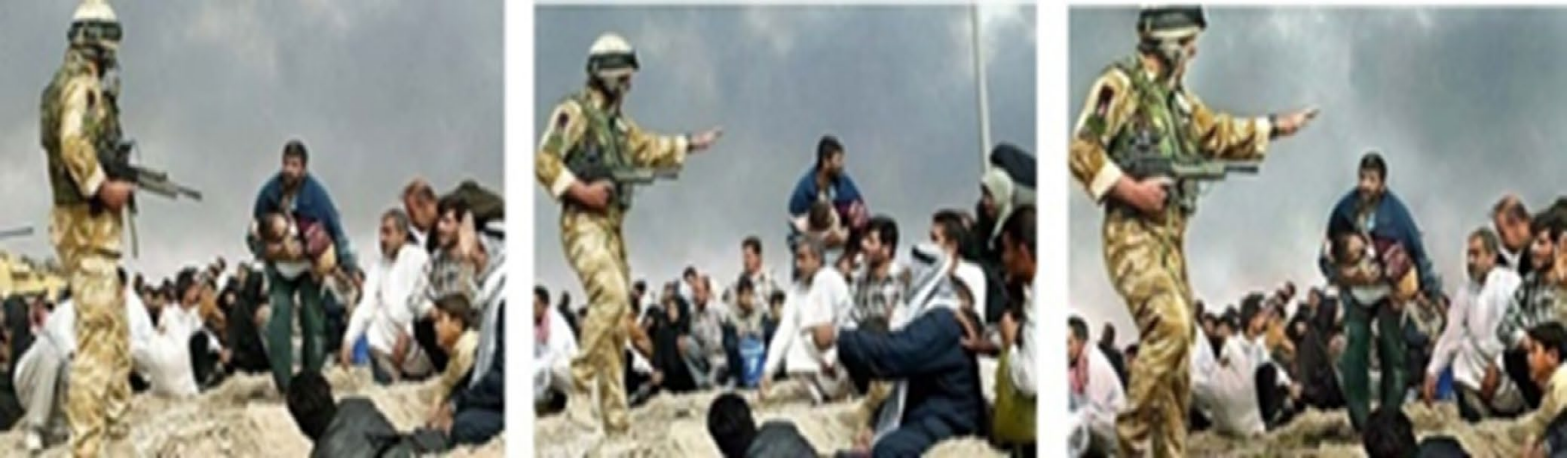
Example images for splicing forgery.

Forgeries in photographs typically take one of three forms: copy‐and‐move, cut‐and‐paste, or erasure. In contrast to copy‐move, which is cutting out and pasting an image region into another frame, the erasing approach requires cutting off an area and replacing its pixels with those from the backdrop. Cut‐paste forgery is combining an element from one image into another. Aiming to rebuild public confidence affected by photo changes and fraud, many studies have investigated ways to handle the problems presented by such image manipulations. These methods have greatly helped develop digital picture tamper detection systems. This work especially targets picture‐splicing forgeries. Because of its link with public and social media information, picture splicing is a common technique of image editing that one encounters in daily life. This sort of alteration involves copying a bit from another photo, which makes identification challenging without obviously differentiating the altered area from the original. Sometimes, post‐processing spliced images minimizes apparent differences, complicating the detecting process. Advanced study in this area is especially crucial given the difficulty of splicing forgery detection relative to other picture‐altering techniques. Figure 1 shows a splicing forgery with the original, altered, and related ground truth photos side by side with white patches denoting altered locations. The changes are evident even under close study; nonetheless, this emphasizes how challenging it is to find such changes with only one's eyes.

Two main categories define several suggested solutions for splicing forgery: conventional techniques and deep learning‐based substitutes. Traditional techniques can rely on traits like noise consistency and color filter array (CFA) artifacts that define modified areas from unspoiled ones [7]. These handcrafted characteristics do, however, have several drawbacks, including less generalizability over several attack forms and less robustness. Contrarily, convolutional neural networks (CNNs) have gained considerable notoriety in computer vision due to their exceptional performance in visual feature extraction [8]. CNN‐based algorithms for image forgery detection have exploded in development as they can dynamically collect intricate image features, which makes them a potent tool for spotting altered content. As a result, many CNN‐based forgery detection systems have surfaced recently and clearly outperformed more traditional approaches. While current techniques concentrate on finding variations between tampered and untampered areas, they sometimes ignore the varied widths of tampered areas among several produced photos, therefore affecting precision [9]. Earlier studies, including DeepLab v2 [3], presented the Atrous Spatial Pyramid Pooling (ASPP) module [10], which efficiently gathers multi‐scale information and supports object segmenting across varying diameters. Taking this a step further, we integrated the ASPP module into the forgery detection system to enhance the segmentation of manipulated areas to many levels. However, the ASPP module is insufficient to address the problem independently.

Recent splicing forgery localization research still leaves several important issues unresolved, which, if answered, might make a major impact. Problems arise, for example, when trying to guarantee resilience in the face of post‐processing techniques like resizing, blurring, and compression, all of which might mask splicing evidence. Detection algorithms struggle to identify spliced sections that blend seamlessly with their surroundings, making improving border accuracy in complex backdrops an additional critical issue. Moreover, since contemporary techniques depending on low‐level features cannot detect more complex forgeries, enhancing feature representation is crucial. Moreover, as models' applicability is restricted when tested on unseen real‐world datasets, cross‐dataset generalization must be improved. Lastly, reducing computational complexity is a top focus since it makes splicing detection methods more scalable and readily accessible for real‐time applications, including deployment on web platforms and mobile devices. Resolution of these problems would significantly advance the splicing forgery detection and localization field.

Many fundamental problems in splicing forgery localization are solved by the proposed CMNV2‐UNet paradigm. Using multi‐scale convolutional layers and MobileNetV2 bottleneck modules, one can easily extract pertinent characteristics from input photos. This captures fine‐grained details over numerous layers, allowing the model to depict features effectively. Second, by concentrating on the most crucial channels required for correct fraud detection, channel attention techniques raise the capacity to analyze local and global settings. Thirdly, the model design combines several convolutional layers to preserve critical visual qualities while limiting post‐processing distortions, including color change, blurring, and compression. Fourth, convolutional transpose layers up‐sample feature maps while maintaining spatial precision, assuring that tampered areas are precisely detected and boosting the splicing localization accuracy.

Finally, the model scales and strengthens rather well by combining conventional methods of deep feature extraction with Canny edge detection. This hybrid approach preserves computational efficiency and allows the model to find splicing forgeries over numerous image datasets. This paper provides a network design that includes U‐Net with MobileNetv2 as its backbone to solve the challenges discovered. The advanced characteristics of MobileNetv2 [11], together with the qualities of the U‐shaped convolutional structure, form this proposed network. By means of ablation research, the network's structure was optimized, hence improving performance on DEFACTO [12], In the Wild [13], and CASIA [14] datasets over current methods. Our work makes the following main contributions.

- To extract strong features at several levels, the model aggregates conventional edge detection approaches (Canny) with deep learning methods (convolutional layers and bottleneck modules). By capturing both fine‐grained and high‐level picture information, our CMNV2‐UNet technique enhances the identification of splicing artifacts.
- Integrating channel attention methods helps the model to concentrate on salient elements in several visual areas. This model increases localization accuracy, particularly when differentiating altered parts from the background.
- By using a deep convolutional network with up‐sampling layers (convolutional transpose), the model efficiently manages typical post‐processing methods, including blurring and compression. This model guarantees exact splicing detection even in compressed or changed photos.
- The model shows a strong localizing ability for post‐processing techniques such as blurring, compression, or noise addition for forgeries. This is vital for practical uses where forgeries are sometimes altered to hide themselves.

The section on Related Work then explores current forgery detection techniques and notes their flaws. The comprehensive construction of the hybrid model follows the Proposed Method, emphasizing its accuracy and efficiency. After the model's evaluation of benchmark datasets in the section on Experiments and Results, the section on Discussion covers potential developments through data interpretation. Finally, the Conclusion and Future Work section notes the contributions and offers guidelines for the following research.

## 2 RELATED WORK

The frequency of manipulated photographs and false information is rising as image editing technology develops. With an eye toward two main tasks, forgery classification and forgery localization, detecting altered images has grown to be a vital area of research. While localization works to identify the areas that have been changed, forgery classification seeks to ascertain whether an image has been manipulated. One can observe the localization of created areas as a type of semantic segmentation. Two important groups of recently proposed splicing forgery detection tools are standard feature extraction methods and approaches using deep learning, as compared in Table 1. Among the anti‐forensics methods offered by Fang and Stamm is a generative adversarial network (GAN) approach that incorporates EXIF‐Net, Noise print, and Forensic Similarity Graphs [15]. This technique creates realistic and self‐consistent forensic traces that appear real during analysis by training a convolutional generator in two steps. While keeping good visual quality in the assaulted photographs, the approach was tested against many forensic databases, including the Columbia, Carvalho (DSO‐1), and Korus databases, revealing a significant loss in detection accuracy and localization ability. Another transformer‐style network, presented by Liu et al. [16] to efficiently captured spliced traces in both the RGB and noise domains, improving image forgery localization. The obtained features were fused using an Attention‐aware Hierarchical‐feature Fusion Module (AHFM), which combined hierarchical features from both domains into one feature map. Several publicly available datasets, such as NIST16, CASIA v1.0, IMD20, and Realistic, were used to test the technique. TBFormer outperformed current state‐of‐the‐art techniques with an *F*_1_ score of 83.4% on NIST16, AUC values of 95.5% on CASIA v1.0, and 86.3% on IMD20. In another study, a Progressive Feedback‐Enhanced Transformer (ProFact) network was presented by Zhu et al. [17]. The method used a coarse‐to‐fine mechanism whereby an initial branch network creates a coarse localization map, which is subsequently adapted to the early transformer encoder levels. On the Columbia dataset, the ProFact network attained an *F*_1_ score of 84.5%; on CASIA v1, it obtained 56.4%.

**TABLE 1:** Deep learning‐based splicing forgery detection and localization methods.

Ref. year	Features	Localization	Dataset	Accuracy	Limitations
Fang and Stamm [15]	GAN	GAN‐based Forgery Detection & Localization	Columbia, DSO‐1, Korus	68%	High computational complexity due to multi‐network training; challenging for real‐time deployment
Liu et al. [16]	Two Branch Transformer	Dual‐Branch Forgery Detection & Localization	NIST16, CASIA v1.0, IMD20	83.4%, 95.5%, 86.3%	Increased complexity due to dual‐branch architecture; challenges in real‐time deployment
Zhu et al. [17]	Progressive Feedback‐Enhanced Transformer	CSPM, Coarse‐to‐Fine Mechanism	Columbia, CASIA v1, NIST16, DSO‐1	84.5%, 56.4%	Increased complexity and computational cost due to feedback mechanism and transformer architecture
Shipra Jain and Durgesh Singh [18]	VGG16 Unet	Patch Extraction	NIST, CASIA v2	98.08%	High complexity and need for high computational resources, limiting scalability in real‐time applications
Tan et al. [19]	Dual Encoder Network (DAE‐Net)	ECA Module	CASIA v2, Spliced COCO, IMD2020, NC16 Splicing	97.9%	Increased complexity due to multi‐scale feature fusion and ECA modules; potential challenges in real‐time applications.
Li et al. [20]	Multiresolution Hybrid Features	Tamper‐Guided Dual Self‐Attention (TDSA) Network	CASIA v1.0, Columbia, NIST16, IMD2020	93.80%	Higher computational demands due to multiresolution feature fusion and TDSA module complexity
Ghannad and Passi [21]	U‐Net with Grasshopper Optimization	U‐Net (GOA)	CASIA v1.0, CASIA v2.0	95.31%	Extensive training required, potential challenges in real‐time applications
Beijing et al. [22]	Quaternion Two‐Stream R‐CNN with ARPN and FPN	Pixel‐level color splicing localization	CASIA v1.0, CASIA v2.0, DVMM	77.87%	High computational cost, potential challenges in real‐time processing
Li et al. [23]	Multi‐Scale Guided Learnin	Self‐Attention Mechanisms, ResNet‐50	CASIA v1.0, Columbia Uncompressed, DSO‐1	–	High computational complexity, extensive training data required
Gu et al. [24]	DFSAM, multitask learning	Frequency decomposition	CASIA v1.0, CASIA v2.0, IMD2020, Columbia, Carvalho, Coverage	76.98%	High computational requirements, challenges in real‐time applications
Guo et al. [25]	Multi‐branch feature extractor	Hierarchical fine‐grained	CASIA, NIST16	76.98%	Complex hierarchical classification, extensive training required
Shi et al. [11]	MobileNetV2 with dual‐stream network (RGB and SRM)	Feature map‐based segmentation	CASIA v1.0, Columbia	75.08%	Reduced performance under low JPEG compression
Mareen et al. [26]	GAN‐based fusion, CAT‐Net, Noise print	Pixel‐level forgery localization	IMD2020, NC2016	86.90%	Struggles with unseen datasets, high computational requirements
Kwon et al. [27]	RGB and DCT streams, pretrained on double JPEG detection	Pixel‐level forgery localization	CASIA‐v2, IMD2020	93.93%	Performance drops for non‐JPEG images due to lack of compression artifacts
Rao et al. [28]	CRF, CNN	Attention Maps	DSO‐1, Coverage	62.40%	High computational cost due to CRF integration
Ding et al. [29]	Dual‐channel U‐Net, High‐pass filter, multi‐scale dilated convolution	Pixel‐level forgery localization	CASIA v2.0, Columbia	86.67%	High computational cost due to multi‐feature fusion
Chen et al. [30]	LSTM, Rotating Residual Units	Pixel‐level forgery localization	NIST16, COVERAGE, CASIA	98.90%	High computational cost due to hybrid feature extraction
Abhishek and Jindal [31]	VGG‐16, Semantic Segmentation	Pixel‐level forgery localization	GRIP, DVMM, CMFD	98.48%	Reduced performance on compressed and rotated images
El Biach et al. [32]	ResNet50, U‐Net, False‐Unet	Pixel‐level forgery localization	CASIA v2.0, NIST'16	81.56%	Reduced performance in compressed images and high computational complexity

Jain and Singh [18] presented VGG16Unet, a passive, effective model intended for localizing picture splicing forgeries. The model used an encoder‐decoder design whereby the U‐Net‐inspired architecture acts as the decoder, and the VGG16 pre‐trained network acts as the encoder. Tested rigorously on two often‐used datasets, NIST and CASIA v2, the model exceeded earlier methods. Apart from an IoU score of 0.9754, the VGG16Unet proved resilient in precisely localizing tampered areas on the NIST dataset with an accuracy of 0.9808 and a recall of 0.9860. A dual encoder network (DAE‐Net) intended to detect picture splicing forgery is proposed by Tan et al. [19]. Combining a VGG‐16 pre‐trained network for the backbone encoder and a residual network for the branch encoder, the network uses a dual encoder structure. The Efficient Channel Attention (ECA) module included in the DAE‐Net acts as an auxiliary mechanism to concentrate on tampered features, hence enhancing multi‐region tampering identification accuracy. With precision rates ranging from 97.9% on Spliced COCO to 90.6% on CASia v2, the DAE‐Net showed remarkable performance on datasets like CASia v2, Spliced COCO, IMD2020, and NC16 Splicing.

Li et al. [20] presented a method based on the Tamper‐Guided Dual Self‐Attention (TDSA) network paired with multiresolution hybrid features to identify and localize image counterfeiting. Combining multiresolution hybrid properties from RGB and noise streams helps the network efficiently discover visual and compression consistency errors. The evaluation of this technique included four public datasets: CASIA v1.0, Columbia, NIST16, and IMD2020. Resilient throughout several picture changes, the approach demonstrated improved performance with an *F*_1_ score of 93.8% on NIST16 and 90.8% on Columbia. Significant accuracy was obtained with the approach by Ghannad and Passi [21], including U‐Net using the Grasshopper Optimization Algorithm (GOA). The U‐Net model optimized by GOA attained an accuracy of 95.31% on the CASIA dataset, hence exceeding other models examined in the research.

In their proposal, Beijing et al. utilized a quaternion two‐stream R‐CNN network to localize color picture splicing at the pixel level [22]. The model can extract multi‐scale features in addition to ARPN (attention region proposal network) to focus on essential areas and FPN (feature pyramid network) based on QResNet. This method performed exceptionally well on multiple datasets, including the CASIA v2.0, CASIA v1.0, and DVMM standard forgery datasets, with an F‐measure of 0.7787. Li et al. introduced a new architecture for deep learning that uses a fully convolutional network (FCN) that integrates self‐attention mechanisms with a multi‐scale directed learning technique [23] for picture splicing localization. The model outperformed state‐of‐the‐art models by training on the CASIA v2.0 dataset and testing it on the CASIA v1.0, Columbia Uncompressed, and DSO‐1 datasets, especially regarding the *F*_1_ score and Matthews Correlation Coefficient (MCC).

Using a frequency‐based technique mixed with multitask learning and self‐attention mechanisms, a new deep learning model termed FBI‐Net was presented by Gu et al. [24] for image forgery localization. A fully convolutional encoder‐decoder network processes the picture components that FBI‐Net has broken down into low‐frequency and high‐frequency parts using the Discrete Cosine Transform (DCT). By a wide margin, FBI‐Net outperformed existing approaches with an average Intersection over Union (IoU) of 70.99% and an *F*_1_‐score of 76.08% when tested on six benchmark datasets, including CASIA v1.0, CASia v2.0, and IMD2020. HiFi‐Net is a unique hierarchical fine‐grained formulation developed by Guo et al. [25] to detect and locate image counterfeiting. HiFi‐Net's multi‐branch feature extractor helps classify fine‐grained forging properties by processing images at many resolutions, enhancing the detection and localization of produced synthetic areas. On several benchmarks, including CASIA, NIST16, and the freshly constructed HiFi‐IFDL dataset, the approach performed better with an average IoU of 70.99% and an *F*_1_‐score of 76.98%.

MobileNetV2, along with a dual‐stream network including an RGB stream and Spatial Rich Model (SRM) stream, was developed by Shi et al. [11] as a lightweight image splicing tampering localization method. The dual‐stream technique allowed the model to extract both edge texture features and noise consistency features between tampered and natural areas. Combining the two data sets with an upgraded Convolutional Block Attention Module (CBAM) enhanced the model's sensitivity to essential features. With an *F*_1_‐score of 0.7508 and an MCC‐score of 0.7294 on CASIA v1.0, the method achieved superior localization accuracy compared to numerous contemporary methods. Mareen et al. [26] combined the output of neural networks, including CAT‐Net and Noiseprint, with a generative adversarial network (GAN) architecture. The product was a robust system that was able to find forgery traces in several databases, including IMD2020 and NC2016. By using the complementing characteristics of every various detective technique, this fusion considerably increases performance.

CAT‐Net was built as an end‐to‐end, fully convolutional neural network to identify and label spliced areas in photographs correctly. The algorithm investigated visual and JPEG compression concerns by fusing the RGB and DCT streams. The accuracy of detecting altered areas is greatly enhanced by pretraining the DCT stream on double JPEG detection. CAT‐Net proposed by Kwon et al. [27] displayed strong localizing spliced region outperformance of current models, with an accuracy of 93.93% on the double JPEG detection test and more of up to 93.87% on datasets like CASIA‐v2 and IMD2020.

Conditional random fields (CRF) combined with attention models inside convolutional neural networks (CNNs) proposed by Rao et al. [28] showed great promise. The multi‐semantic CRF‐based attention model generates attention maps highlighting boundary transition artifacts produced by forgeries, hence improving feature extraction. For instance, the proposed model obtained 62.4% on the DSO‐1 dataset. Still, there are challenges, including enormous computing complexity, particularly in the stages of feature extraction and training. In recent developments in photo splicing detection, convolutional neural networks (CNN) proposed by Ding et al. [29] have shown promising performance in exactly localizing produced areas. A novel method for pixel‐level photo tampering localization in the dual‐channel U‐shaped network DCU‐Net was presented. The model consists of two fusion phases: one between multi‐scale dilated convolution features and another between the deep features derived from both channels. Experimental data on the CASIA dataset reveal the great accuracy and durability of the model based on an *F*_1_‐score of 86.67%.

One notable approach proposed by Chen et al. [30] captures traces of manipulation by combining long‐short term memory (LSTM) with resampling approaches using hybrid features with semantic reinforcement networks (HFSRNet). Pixel‐level accuracies up to 98.9% and *F*_1_ ratings as high as 0.918 on NIST16 show the method's efficacy in experimental evaluations on the NIST16, COVERAGE, and CASIA datasets. Effective pixel‐level categorization and localization of generated regions are made possible by a hybrid method integrating deep convolutional neural networks (DCNN) with semantic segmentation proposed by Abhishek and Jindal [31]. The method exhibited excellent accuracy—an overall accuracy of 98.48% and an Intersect over Union (IoU) score of 91.14% on datasets comprising GRIP, DVMM, and CMFD. Particularly for copy‐move forgery detection and splicing, convolutional neural networks (CNNs) have tremendously developed the field of visual fraud detection. Combining the ResNet50 encoder with a U‐Net‐based decoder, False‐Unet was proposed by El Biach et al. [32], which builds pixel‐wise binary masks, effectively localizing produced fake areas in images. On CoMoFoD, the model scored *F*_1_‐values of up to 81.56% and did rather well on many datasets, including NIST's 16.0 and CASIA v2.0.

## 3 MATERIALS AND METHODS

**FIGURE 2:**
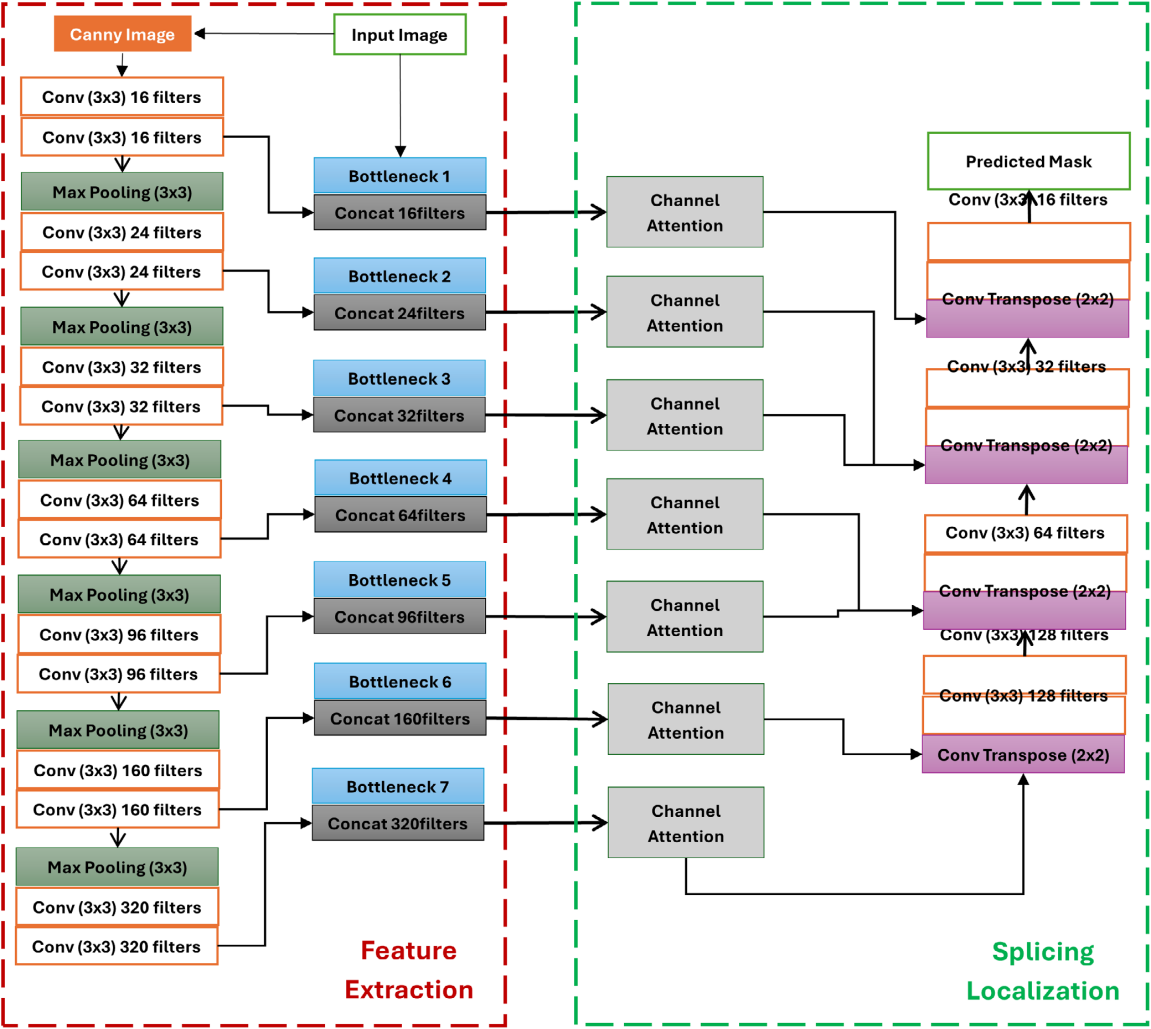
Overall proposed splicing forgery localization model.

A detailed explanation of the proposed CMNV2‐UNet model based on the MobileNetV2 architecture has been displayed in Figure 2. Section 3.1 provides an overview of the model's structure. The procedure for feature extraction is covered in Section 3.2, while the identification of splicing forgery is addressed in Section 3.3. Section 3.3 concludes by providing more details about the multi‐scale supervised learning technique used to optimize the model's training and improve the localization accuracy of the manipulated regions.

### 3.1 Overall proposed architecture

The two parts of the suggested MSU‐Net—feature extraction and splicing forgery localization—are shown in Figure 2. Figure 2 demonstrates that the proposed MSU‐Net model's feature extraction module consists of the Edge stream and MobileNetV2. It uses the canny operator and MobileNetV2 to produce edge information for a specific picture. As shown in Figure 2, the input picture and the obtained results are combined to form the inputs for the edge stream.

The suggested CMV2‐UNet model enhances picture splicing forgery detection and localization by utilizing MobileNetV2 as its feature extraction backbone. A Canny edge detection step emphasizes image boundaries; the design begins with two convolutional layers, each containing 16 filters, which extract fundamental information from the input image. Following this are several bottleneck layers using depth‐wise separable convolutions, which enable minimal computational cost while effectively extracting features. These bottlenecks capture complicated information at several abstraction layers, steadily increasing the filter count—from 16 to 320. The model combines a channel attention mechanism after each bottleneck to prioritize important channels, therefore enhancing the focus on pertinent features for forgery detection. Finally, the splice localization stage uses transposed convolution layers to up‐sample feature maps, thereby enabling the exact prediction of tampered areas in the image and producing an output mask that marks the areas of tampering exactly. This complete series emphasizes how exactly and successfully MobileNetV2 should be implemented in the proposed architecture for splicing false localization.

### 3.2 Feature extraction

Left to right, in Figure 2, you can see the feature extraction module's two streams: the edge stream and the RGB stream. The edge stream is responsible for learning features independent of semantics. In contrast, the RGB stream learns features relevant to semantics, such as color and texture, so the model can grasp what the image is trying to convey. Using semantically related features extracted from the RGB stream makes it impossible to accurately detect post‐processed altered areas, such as altered boundaries, or contrast different portions of a fabricated image. The RGB stream, on the other hand, records data about semantics.

**FIGURE 3:**
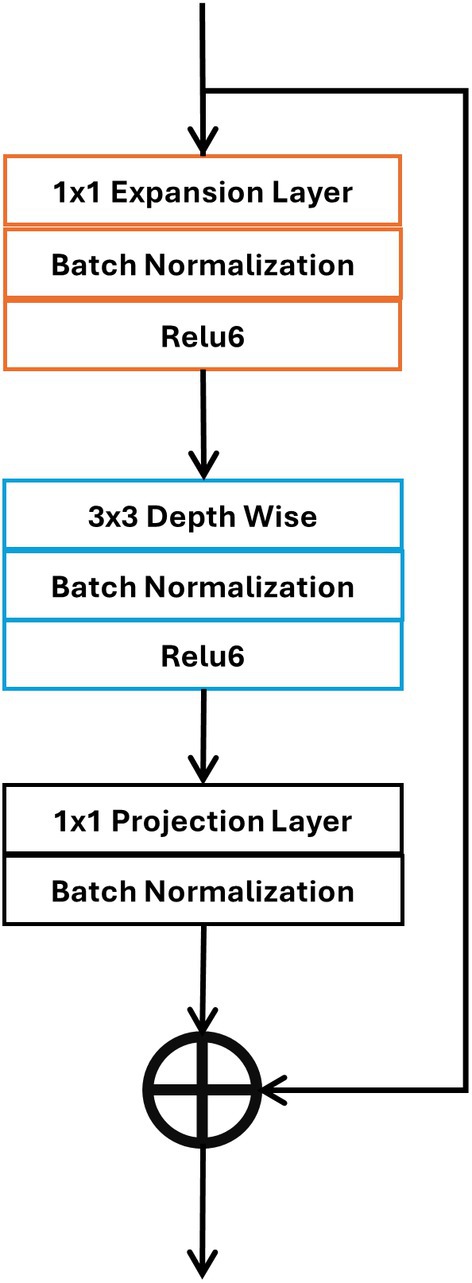
Framework for feature extraction block (Bottleneck residuals using MobileNetV2).

Consequently, it incorrectly identifies altered area edges because it views picture splicing forgery as a semantic segmentation difficulty. To combat this, we develop an edge stream that uses the Sobel operator to improve the accuracy of edge recognition for fake images. With the help of the edge stream, the model can effectively gather image structural information, which enhances the boundary's perception. Take the RGB picture as an example; it will be the Input of the RGB stream. Input edge refers to the inputs of noise edge stream, which is calculated according to:

(1) $${\text{Input}}_{\text{edge}}=\lambda \times \text{Input}+\left(1-\lambda \right)\times {\text{Input}}_{\text{canny}}$$

where Input_Sobel_ represents the outputs of the RGB image passing through the Canny operator, λ ∈ (0, 1) is positive weight. We will conduct a comparative experiment on the values of λ in the experimental results section. Each feature extraction module's branch has seven feature extraction blocks, which produce features at varying depths (shallow to deep), as illustrated in Figure 2. Splicing forgery localization receives the combined result of the feature extraction module. Splicing forgery localization module output is a seven‐level, shallow‐to‐deep concatenation of feature extraction block output at the relevant point in each branch. Figure 3 shows the MobileNetV2 bottleneck residual block, an essential part of the architecture that integrates the flexibility of residual learning with the efficiency of depth‐wise separable convolutions. In feature extraction and dimensionality management of the block, each of the three phases—expansion, depth‐wise convolution, and projection—has a particular function.

The first stage of bottleneck is to expand the dimension of the input tensor image. Given an input tensor *X* of dimension *H* × *W* × *C*, where *H*, *W*, and *C* represent the height, width, and number of channels, respectively, the expansion layer applies a 1 × 1 pointwise convolution to expand the amount of channels by a factor typically 6 [33]. mathematically, the transformation is:

(2) $$\begin{array}{c} X^{\prime }=\text{Conv}\;1\times 1\left(X\right) \end{array}$$

Modern neural networks depend heavily on depth‐wise convolution, particularly in feature extraction techniques where computing efficiency is critical. Its importance stems from its capacity to separately process every channel of input, extracting necessary spatial characteristics free from the computational load related to conventional convolutions. Depth‐wise convolution is essential in designs such as MobileNetV2 to preserve a lightweight structure while nevertheless allowing great performance in picture classification, object identification, and other applications. The residual block and projection layer are fundamental components that cooperate to maintain efficient computations and enhance performance in deep learning architectures, including MobileNetV2. Usually used to either shrink or expand the number of channels, the projection layer is 1 × 1 convolution. Compression of the feature maps produced by depth‐wise convolutions depends critically on this layer, hence regulating the dimensionality and preserving computational efficiency [34]. Within residual blocks, especially bottleneck residual blocks, the projection layer guarantees that features transfer back to the original dimensions, hence enabling residual connections. Avoiding the vanishing gradient issue in deep networks depends critically on these residual connections, which skip intermediate levels. Without adding major computational expense, the model delivers superior gradient flow and deeper network training by conserving the information from older layers and mixing it with newly learned features, hence producing more accurate feature extraction and enhanced general performance. This effective feature extraction qualifies for such designs for real‐time applications on devices with limited resources.

### 3.3 Splicing forgery localization

Fusion of the multi‐level features acquired in the preceding stage is the first order of business. In the feature extraction module, four feature recovery blocks go from shallow to deep to recover the dimensions of the fused features. The topic of this section is how to identify and pinpoint the splicing spots. The U‐Net's ability to recognize objects of varying sizes is enhanced by its multi‐scale design, which allows input information to be immediately propagated across its levels [35]. Extending this, information is combined across several levels using a hierarchical feature fusion approach. This method combines shallow and deep features to help keep important semantic information that deep convolutions could otherwise lose. First applied to each feature level, a channel attention mechanism then follows a 1 x 1 convolution to guarantee the preservation of vital information across the network.

**FIGURE 4:**
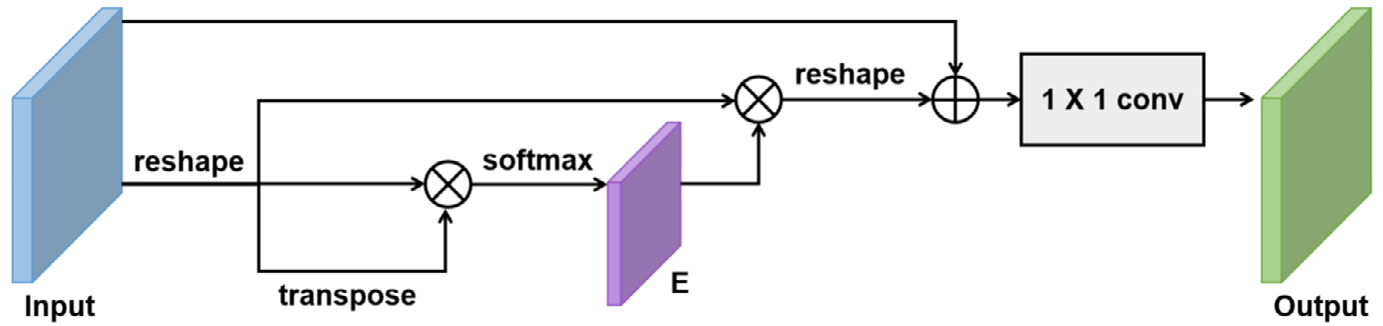
Framework for Channel attention mechanism for feature fusion.

We use feature fusion at several phases and the termination of the feature extraction branches to capture as many signs as possible of manipulating forgeries across several scales and increase the sensitivity of the model to changed areas. While adding pertinent feature mappings, our hierarchical fusion approach combines multi‐level feature representations. This method thus facilitates the extraction and preservation of more complete multi‐scale global contextual information, hence enabling the model to better locate forgery areas by using knowledge from several scales. We implemented a DANet [36] channel attention module, whose structure is depicted in Figure 4, in every layer of hierarchical fusion. The final feature of each channel is a weighted sum of the features from all channels and the original features, denoted by O and O ∈ R^*C*×*H*×*W*^:

(3) $$\begin{array}{c} O_{j}=I_{j}+\gamma \;\sum_{i=1}^{C}\left(E_{\mathrm{ji}}.I_{i}\right) \end{array}$$

where *I* ∈ RC × *H* × *W* is the features obtained from the feature extraction module, γ is a learnable scale parameter with an initial value of 0, *C* is the number of channels, and *E*_*ji*_ denotes the influence of the ith channel on the *j*th channel:

(4) $$\begin{array}{c} E_{\mathrm{ji}}=\frac{\exp\left(I_{i}.I_{j}\right)}{\sum_{i=1}^{C}\exp\left(I_{i}.I_{j}\right)} \end{array}$$

The outputs of the last feature recovery block are received by a segmentation head and a transposition head, respectively, as shown in Figure 2. The segmentation head produces the output that can accurately identify the areas that have been tampered with. The channel attention mechanism, as shown in Figure 4, focuses on the most instructional channels inside a feature map to improve the network's ability to prioritize significant features. It uses a transpose operation first to reshape the input feature map, therefore enabling a pairwise interaction between channels. These interactions then produce attention weights by means of a SoftMax function. The original feature map is scaled using these weights, therefore emphasizing significant channels and suppressing less pertinent ones. The reshaped and weighted features are convolutionally layered (1 × 1) to provide the output, where critical channels have been amplified, hence enhancing the model's focus on important details in tasks such as picture classification and splicing forgery detection. Focusing on significant channel information, this approach improves feature representation quite successfully. The channel attention mechanism, as shown in Figure 4, focuses on the most instructional channels inside a feature map to improve the network's ability to prioritize significant features. It first reshapes the input feature map using a transpose operation to enable pairwise interactions across channels. These interactions produce attention after a SoftMax function. By adjusting these weights, the original feature map can be scaled to highlight more essential channels while reducing the visibility of less important ones. The reshaped and weighted features are convolutionally layered (1 × 1) to provide the output, where critical channels have been amplified, hence enhancing the model's focus on important details in tasks such as picture classification and splicing forgery detection. Focusing on significant channel information, this approach improves feature representation quite successfully.

## 4 EXPERIMENTS AND RESULTS

To assess how well the suggested model works, this part runs comprehensive tests on several publicly accessible datasets. We begin by doing ablation research to ensure each module functions as intended. Following that, we compare the suggested approach to state‐of‐the‐art methods, such as deep learning models that have recently been established and methods that extract features manually. Moreover, the proposed method is tested for its resilience against several threats, such as JPEG compression, Gaussian blur, and Gaussian noise attacks. Lastly, we evaluate the proposed model's execution time compared to similar models.

### 4.1 Experimental settings

#### 4.1.1 Datasets

The CASIA v2.0 dataset was initially divided 80/20 into training and test sets to conduct the ablation experiment using the suggested model. In addition, the Defacto‐Splicing forgery dataset was utilized for training purposes [37]. Data sets, including Columbia, NIST16, IEEE IFS‐TC, and In‐the‐Wild, were used for testing and comparison. These databases include splicing, copy‐move, and object removal, among several kinds of altered photos. However, only the splicing photos from these datasets were employed in the training and testing stages since the model concentrates especially on splicing forgery localization. For further information on these datasets, see Table 2. Using CASIA v2.0 and Defacto‐Splicing for training and testing in the ablation research (Section 4.2), we evaluated performance on four test datasets: Columbia, NIST16, In‐the‐Wild, and other datasets (Section 4.3).

**TABLE 2:** Detailed information of benchmark datasets used to evaluate the model's effectiveness.

Sr.	Dataset	Image types	No. images	Spliced samples
1	CASIA v2.0 [14]	TIFF, JPEG	5123	1828
2	Columbia [38]	BMP, TIFF	180	180
3	NIST16 [39]	JPEG	564	288
4	In the Wild [13]	PNG	201	201
5	Defacto‐Splicing [37]	TIFF	10,800	10,800
6	IEEE IFS‐TC [40]	PNG	2124	479

For a dataset mismatch analysis, we trained the model on one dataset (CASIA 2 and DEFACTO) and then tested it on many datasets (Columbia, NIST16, In the Wild, IFS‐TC and CASIA) without making any changes to see how well our method could work with all of them. This cross‐dataset validation guarantees that the model is learning actual splicing traces instead of artifacts particular to a dataset. The results show that our method works well across multiple datasets, proving that it can find splicing frauds in a wide range of image distributions.

#### 4.1.2 Evaluation measures

Using values between 0 and 1, this study assesses the Area Under the Receiver Operating Curve (AUC) and pixel‐level *F*_1_ score. Above everything else, higher values signify superior model efficiency. The *F*_1_ score comprehensively evaluates the model's performance by combining Precision and Recall. Both recall and precision measure the percentage of tampered pixels that are correctly classified relative to the total number of altered pixels. What follows is the formula for calculating *F*_1_, Precision, and Recall:

(5) $$\begin{array}{c} \text{Precision}=\frac{\mathrm{TP}}{\mathrm{TP}+\mathrm{FP}}\; \end{array}$$

(6) $$\begin{array}{c} \text{Recall}=\frac{\mathrm{FP}}{\mathrm{TP}+\mathrm{FN}} \end{array}$$

(7) $$F_{1}\;\text{Score}=2\times \frac{\text{Precision}\times \text{Recall}}{\left(\text{Precision}+\text{Recall}\right)}$$

There are three variables: TP for total pixels that have been appropriately classified as tampered, FP for total pixels identified as actual tampered, and FN for the number of pixels classified as genuinely tampered. By plotting the True Positive Rate (TPR) vertically and the False Positive Rate (FPR) horizontally, we may find the area under the receiver operating characteristic (ROC) curve, which is abbreviated as AUC. The following are the calculated AUC, TPR, and FPR:

(8) $$\begin{array}{c} \mathrm{FPR}=\frac{\mathrm{FP}}{\mathrm{FP}+\mathrm{TN}}\; \end{array}$$

(9) $$\begin{array}{c} \mathrm{TPR}=\frac{\mathrm{TP}}{\mathrm{TP}+\mathrm{FN}}\; \end{array}$$

(10) $$\begin{array}{c} \mathrm{AUC}=\int_{0}^{1}\mathrm{TPR}\left(\mathrm{FPR}\right)\text{dFPR}\; \end{array}$$

TN stands for the total number of actual pixels accurately classified.

#### 4.1.3 Implementation settings

Using the PyTorch framework, the CMV2‐Net model was constructed on an NVIDIA A5000 GPU. The training set included input images with dimensions of 384 × 256 and a batch size of 8. The Adam optimizer was employed to provide the best possible results for the model, beginning with a learning rate of 10^−3^ and gradually decreasing to 103. We determined the range of positive values for λ in Equation (1) from 0.1 to 0.9 using the comparative experimental data from Section 4.2. Blurring, inverting, and compression were other approaches meant to reduce data variation. To increase the generalizing capacity of the model over multiple sizes, orientations, and image distortions in segmentation activities, further augmentations, including random cropping, rotation, scaling, and elastic deformation, were done. These techniques improve the localization accuracy of segmentation and enable the strengthening of fine border detection.

### 4.2 Performance of proposed CMV2‐UNet

**FIGURE 5:**
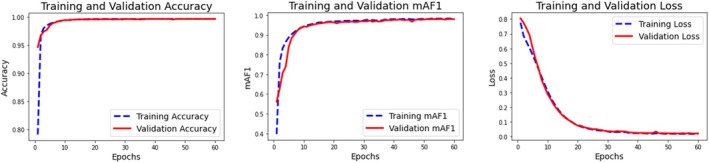
Validation performance of proposed CMV2‐UNet Model.

Emphasizing accuracy, mAF1 (mean average *F*_1_ score), and loss, Figure 5 shows the training and validation performance of the CMV2‐UNet model over 60 epochs. Both training and validation accuracy rise rapidly on the accuracy graph, reaching nearly 98% in the first 10 epochs, suggesting that the model converges quickly with minimal overfitting. Reflecting the great balance between precision and recall across many classes, the center graph, displaying mAF1 scores, follows a similar trajectory and stabilizes at almost ideal values following the early epochs. Finally, the loss graph reveals a sharp drop in earlyraining and validation loss; both curves are tightly matched and level off around zero following the first few epochs. The almost exact behavior of the training and validation measures in all three graphs indicates a well‐regularized CMV2‐UNet model with a low risk of overfitting and high generalizing power.

The CMV2‐UNet model has impressive performance, although there are some little but essential lessons here. One potential disadvantage of its complex architecture, which depends on several criteria, is that it could generate more computational needs in resource‐limited or real‐time environments. Moreover, even if it performs poorly on the validation set, the model can demand adjustments or fine‐tuning to operate effectively on datasets with different distributions or imbalanced classes. Even if the model's *F*_1_ scores and extraordinary accuracy demonstrate, tiny preparation or training data changes may still damage its robustness and hinder its adaptation in particular contexts without enough preprocessing or modification.

### 4.3 Ablation experiments

To assess the complete effect of the CMV2‐UNet, we test the suggested model in three distinct environments. First, extracting features: We conduct comparative experiments after fine‐tuning our method's various extraction branches. Table 3 displays the outcomes of these studies. Networks with one feature extraction branch (e.g., RGB stream, Canny edge stream, or Sobel edge stream) are represented by serial numbers 1, 2, and 3 in the table; networks with feature fusion based on concatenation are represented by serial numbers 4, 5, and 6. Finally, our suggested network is represented by serial number 9, while networks with feature fusion based on the channel attention mechanism are denoted by numbers 7, 8, and 9. Table 3 reveals that out of all the networks, MSUET has the best AUC at 96.56% and the best *F*_1_‐score at 94.37%. Every branch is crucial to the network's functionality, as this demonstrates.

**TABLE 3:** Performance comparison of proposed model in different scenarios.

Sr.	RGB	Edge detection operator		Fusion method	AUC	*F*_1_‐score	FPR
		Canny	Sobel				
1	+	−	−	−	81.13	57.26	0.32
2	+	−	+	−	75.21	47.82	0.28
3	+	+	−	−	79.25	65.71	0.25
4	+	−	−	Concatenation	82.34	57.22	0.21
5	+	−	+	Concatenation	84.18	63.85	0.14
6	+	+	−	Concatenation	82.69	73.54	0.16
7	+	−	−	Channel Attention	80.02	63.65	0.19
8	+	−	+	Channel Attention	85.89	68.38	0.18
9	+	+	−	Channel Attention	96.56	94.37	0.03

The ablation experiment in Table 3 reveals the significance of every element in improving the model's forgery detection performance. To analyze their effects on significant measures, including AUC, *F*_1_‐score, and FPR, we tested many combinations of RGB input, edge detection operators (Canny, Sobel), and fusion approaches. Using just RGB images (row 1), the model achieves a modest AUC of 81.13%, hence showing baseline performance. Edge detection (Canny) (row 3) somewhat enhances the detection capacity of the model with an *F*_1_‐score jump to 65.71% and a lowered FPR of 0.258, thereby highlighting the need to extract finer features from edge details. Moreover, applying concatenation fusion in conjunction with edge detection (row 6) shows a significant improvement that pushes the *F*_1_‐score to 73.54% and lowers the FPR to 0.169, thus stressing the efficiency of fusing multi‐level features for forgery localization.

The most important findings come from including channel attention coupled with edge detection (row 9). This environment obtains the best AUC of 96.56%, an amazing *F*_1_‐score of 94.37%, and a shockingly low FPR of 0.031, therefore indicating the model's capacity to record complex information from the altered areas. Effective focus on relevant properties using channel attention produces better detection and localization capacity. Edge detection allows high accuracy and low false positives since it helps the model focus on both global and local picture information, therefore optimizing the configuration for forgery detection by means of channel attention. Combining Canny edge detection with channel attention maximizes the feature extraction and emphasizes changed locations, thereby considerably enhancing the forgery localization accuracy.

Presenting both AUC (Area Under the Curve) and *F*_1_‐score, the ablation experiment results displayed in the graphs enable one to grasp the performance of a model with sobel and canny weights over different values of λ (lambda). The results show that the AUC and *F*_1_‐score vary as the value of λ increases from 0.1 to 0.9, therefore reflecting variations in the capacity of the model to detect and classify features effectively.

**FIGURE 6:**
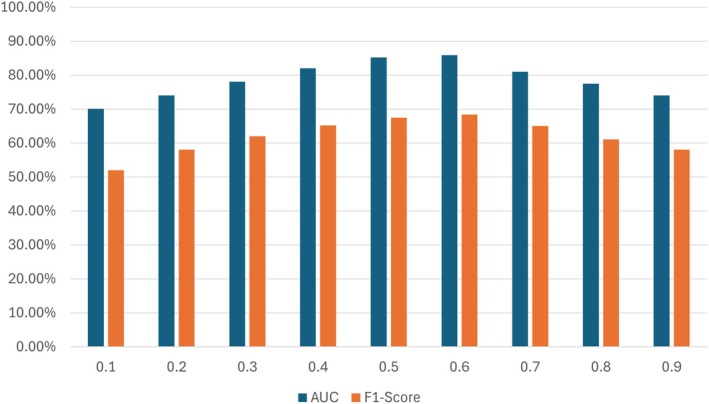
Comparison of model performance on different weights while using sobel in inputs for feature extraction.

As shown in Figure 6 Both the AUC and *F*_1_‐score are rather lower at smaller values of λ (e.g., 0.1, 0.2), suggesting that the model is less effective in these settings. Both measures peak as λ gets close to 0.5 and 0.6; the *F*_1_‐score is about 68.38%, and the AUC is about 85.89%. These results suggest that these values of λ capture the optimal equilibrium for the performance of the model. It suggests that by use of Sobel weights combined with these λ values, more exact edge detection and feature extraction enhance classification and segmentation results. As λ reaches 0.7 and above, the AUC and *F*_1_‐score start to decline, therefore indicating a somewhat performance loss. This would mean that too much reliance on the Sobel operator at higher values of λ could lead to overfitting or reduced generalization capacity, thereby affecting the accuracy of the model predictions. In this experiment, the ideal configuration for picture segmentation tasks is found generally at λ values between 0.5 and 0.6, where the combination of Sobel edge detection and optimal weighting contributes to the highest performance metrics.

**FIGURE 7:**
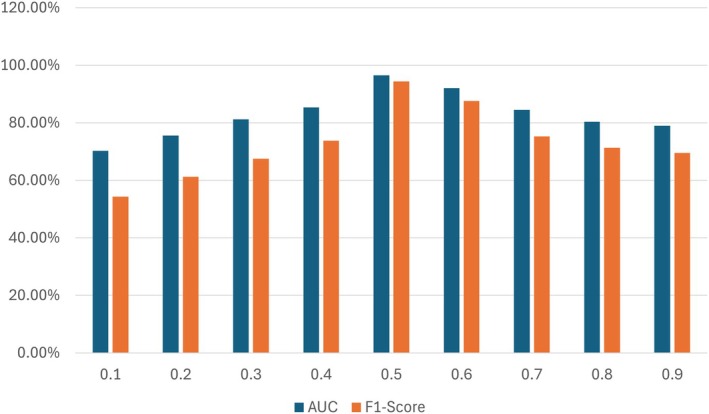
Comparison of model performance on different weights while using canny inputs for feature extraction.

By means of Canny inputs for feature extraction, the graph in Figure 7 contrasts the performance of the model over several lambda values. The AUC and *F*_1_‐score show a clear performance peak at lambda values of 0.5 and 0.6. The AUC exceeding 80% at these values indicates that the model can vary among many classes. Similarly, these sites have the best *F*_1_ score, demonstrating the model's ability to balance recall and accuracy properly. These levels' selected weights of these levels considerably improve the Canny operator feature extracting process. The performance falls at the extremes of the lambda range, 0.1 and 0.9, suggesting that too little or too much focus on features can undermine the model's efficiency. Therefore, the results emphasize the importance of optimizing model performance in this context by changing lambda weights.

### 4.4 Comparison with state‐of‐the‐art methods

Table 4 contrasts several splicing forgery detection techniques using Columbia, NIST16, and In‐the‐Wild datasets. Separately evaluating two metrics—AUC (Area Under the Curve) and *F*_1_‐Score—showcases the model's capacity to discriminate between fake and real images and the balance between recall and accuracy. Of the models, CMV2U‐Net is the one that most obviously outperforms its competitors. It receives the best AUC and *F*_1_ scores on all datasets—88.52% and 82.37% for Columbia, 94.46% and 93.21% for CASIA, 91.20% and 77.65% for NIST16, and 78.25% and 67.61% for In‐the‐Wild. Under many conditions, these results exhibit improved model consistency and resistance in splicing forgery detection. Models such as SATFL and HDU‐Net exhibit poorer performance, particularly with low *F*_1_ scores, therefore stressing issues in precision‐recall balancing. Although they perform somewhat well as CMV2U‐Net, the Forensics OSN and MSU‐Net models fall short. This comparison of both AUC and *F*_1_‐Score reveals that CMV2U‐Net is among the most recent models regarding detection accuracy.

**TABLE 4:** Comparative analysis with recent splicing forgery detection models.

Citation	Method	CASIA		Columbia		NIST16		In the wild	
		AUC	*F*_1_	AUC	*F*_1_	AUC	*F*_1_	AUC	*F*_1_
Chen et al. [41]	MVSS‐Net	81.70	59.60	74.00	60.90	74.60	29.00	66.70	36.70
Wei et al. [42]	HDU‐Net	84.50	73.40	71.70	58.80	76.00	36.50	72.30	48.20
Zhuo et al. [43]	SATFL	63.00	30.30	63.20	43.00	59.20	28.20	60.70	37.80
Wu et al. [44]	Forensics OSN	86.60	69.90	76.70	66.10	79.80	49.90	75.10	50.50
Xu et al. [45]	FE‐PCL	–	–	72.30	58.20	–	–	66.70	46.40
Yu et al. [46]	MSU‐Net	87.00	70.60	80.00	67.60	83.20	50.20	71.50	46.70
Gao and Huang [47]	FP‐Net	–	–	70.30	–	74.20	–	69.60	–
Wu et al. [48]	ManTra‐Net	76.60	–	70.10	–	45.50	–	–	–
Proposed method	CMV2U‐Net	94.46	93.21	88.52	82.37	91.20	77.65	78.25	67.61

Although CMV2‐Net shows remarkable performance gains above other models, it does have some smaller restrictions. One difficulty is its possible sensitivity to differences in the data distribution among several datasets. Although the model works well across datasets such as CASIA, Columbia, and NIST16, its success may somewhat decrease when used with datasets that have notable variations in characteristics or composition, thereby calling for some degree of fine‐tuning. Furthermore, although efficient, the model's architecture can be prone to overfitting should the dataset be too small or lacking diversity—a typical problem with highly parameterized deep learning models. Finally, even if CMV2U‐Net usually produces good AUC and *F*_1_ scores, its inference performance for real‐time applications should be optimized where faster detection could be vital.

### 4.5 Robustness analysis of the proposed method and comparison with the SOTA method

The comparison results in Table 5 underscore the endurance of numerous splicing forgery detection techniques under several post‐processing attacks, such as blurring, noise, and JPEG compression, using CASIA, Columbia, and NIST16 datasets. The proposed CMV2U‐Net approach frequently outperforms previous models, including MVSS‐Net, HDU‐Net, and MSU‐Net, for all attack kinds and datasets. For blurring assaults on CASIA, 89.28% under noise on Columbia, and 92.69% under JPEG compression on NIST16, CMV2U‐Net receives the greatest AUC scores, for example. These findings show its exceptional capacity to preserve detection accuracy even following different post‐processing aberrations.

**TABLE 5:** Robustness analysis with AUC in different post‐processing scenarios using CASIA, Columbia, and NIST16 dataset.

Citation	Method	Dataset	Blurring	Noise	JPEG compression	W/O attacks
Chen et al. [41]	MVSS‐Net	CASIA	80.30	77.20	81.10	81.70
		Columbia	66.80	71.80	70.80	74.00
		NIST16	68.60	74.30	72.20	74.60
Wei et al. [42]	HDU‐Net	CASIA	82.80	77.00	71.40	84.50
		Columbia	71.70	68.20	65.90	71.70
		NIST16	74.60	75.10	73.10	76.00
Yu et al. [46]	MSU‐Net	CASIA	85.30	83.60	83.90	87.00
		Columbia	73.00	71.10	66.10	80.00
		NIST16	80.50	80.50	77.30	83.20
Kong et al. [49]	Pixel Inconsistency	Columbia	79.00	70.00	–	68.00
		CASIA	57.83	52.20	–	56.60
		IEEE IFS‐TC	48.23	41.60	–	45.50
Yadav and Vishwakarma, [50]	DenseNet CNN	CASIA	–	–	91.26	93.82
Proposed method	CMV2U‐Net	CASIA	91.23	92.31	91.56	94.46
		Columbia	87.32	89.28	90.48	88.52
		NIST16	90.36	91.25	92.69	91.20
		IEEE IFS‐TC	93.56	91.48	90.67	95.20

The CMV2U‐Net has certain milder constraints even if it runs efficiently. Its complicated architecture helps to explain its high performance since it requires more processing resources and makes implementation on low‐power devices or real‐time systems difficult. While CMV2U‐Net performs effectively against the studied post‐processing attacks, its performance against more complicated and mixed attack scenarios—e.g., a mix of noise and compression—has not been studied. Therefore, future research is still feasible. Future iterations of the proposed method could maximize the model for speed and resource economy, maybe using pruning or quantization techniques, thereby improving the recommended methodology. Furthermore, the generalizability and adaptability of the model to many real‐world scenarios will be ensured by extending the robustness of the research to take more varied datasets and a greater spectrum of attack combinations.

**FIGURE 8:**
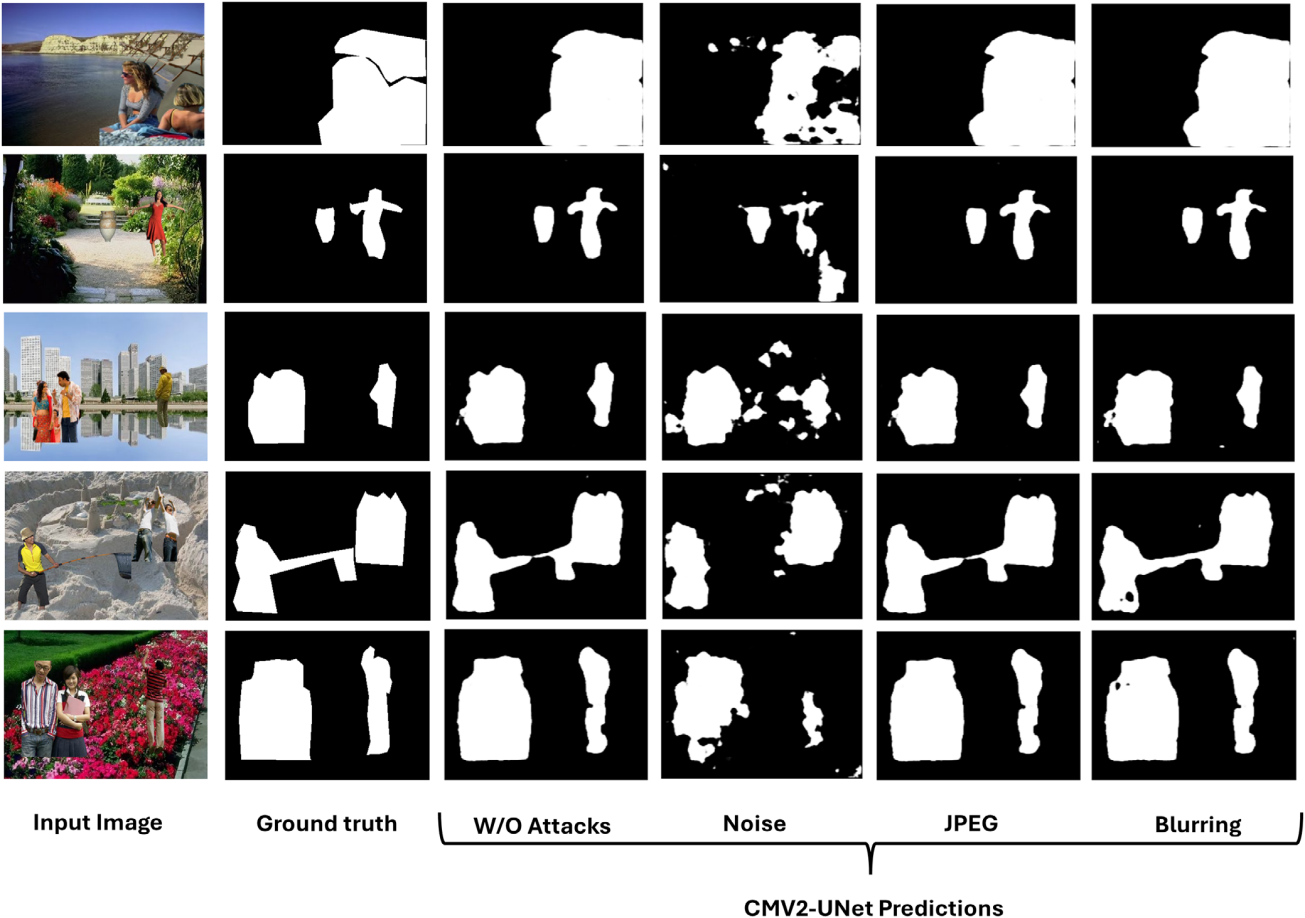
Visual results on unseen samples from the CASIA dataset.

While also exposing some minor shortcomings, the visual findings shown in Figure 8 from the CASIA dataset emphasize the efficacy of the proposed CMV2‐UNet model in many circumstances. As the “Without Attacks” column shows, the model exhibits good segmentation performance without adversarial attacks applied. Under these clean conditions, closely matched ground truth masks reveal the model's capacity to detect and segment items precisely. JPEG compression, blurring, and noise are all unfriendly tasks for the CMV2‐UNet model. Despite some apparent vestiges and lost attributes, the model captures the key elements in the “Noise” column. The JPEG compression and blurring effects highlight the model's potential because segmentation preserves shape and structure, particularly in more crucial items. This demonstrates how effectively the model withstands frequent real‐world distortions, retaining appropriate segmentation even in less‐than‐ideal conditions. However, the method has several limitations. As the third column demonstrates, it is significantly more noise‐sensitive, where forecasts grow dispersed or absent. This suggests that the model controls a particular noise effectively, even if it performs somewhat poorly in high‐noise settings.

Moreover, JPEG compression and blurring occasionally result in lost fine details, particularly in complex or smaller objects with less well‐defined object boundaries. These minor defects could impact applications requiring exact segmentation. Although, in some cases, its sensitivity to noise and some loss of small features point to areas for further work, generally, the CMV2‐UNet model shows outstanding robustness and efficiency in both clean and hostile environments. Its resilience to JPEG compression and blurring makes it somewhat helpful in real‐world scenarios; resolving too significant aberrations will require extra fine grading.

**FIGURE 9:**
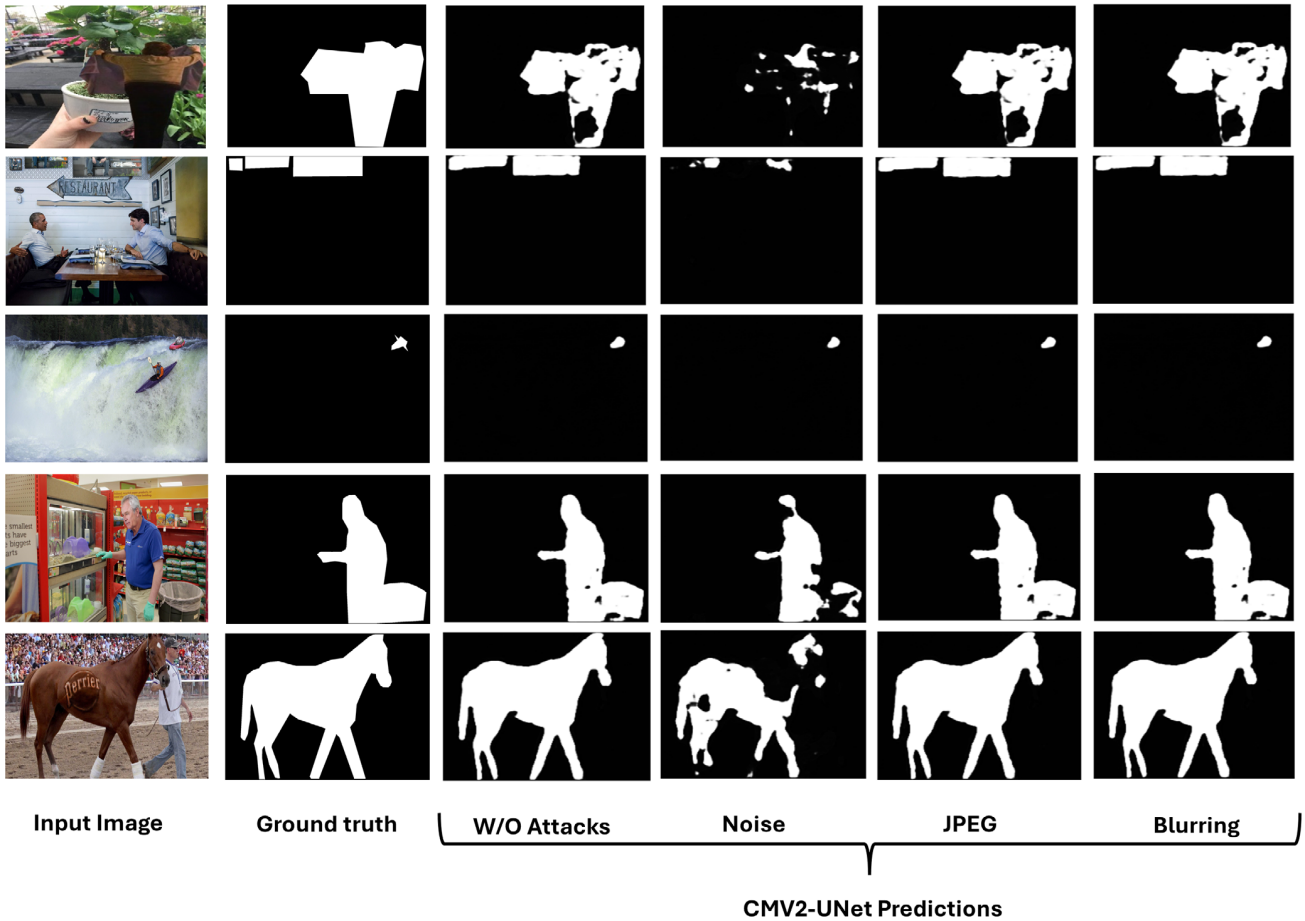
Visual results on unseen samples from In the Wild dataset.

Extracted from the “In the Wild” dataset, Figure 9's visual results show CMV2‐UNet model performance under many conditions, including without assaults and with noise, JPEG compression, and blurring. These results provide more evidence of the approach's advantages and disadvantages in more complex, pragmatic contexts. With the expected masks tightly aligned with the ground truth, the model works well under clean conditions, shown in the “Without Attacks” column. This indicates that the model can effectively separate things, including humans, animals, and surroundings, in more natural, varied settings, hence generalizing to unseen, real‐world photographs. Notable is also the model's robustness under hostile circumstances, including JPEG compression and blurring. In most of these cases, especially with the horse in the last row, the model preserves the form and fundamental features even with distortions. The model performs excellently even in more complex situations (like the man at a store and the horse). This implies that CMV2‐UNet is fit for managing typical picture degradation problems in uncontrolled settings.

Still, there are some restrictions under more demanding circumstances. As the “Noise” column indicates, the model exhibits increased noise sensitivity. For some photographs, particularly with complicated backdrops or moving objects (e.g., the surfer in the third row), the segmentation fails and shows little to no ground truth resemblance. This highlights how difficult the model is to manage noise‐heavy environments, particularly when the backdrop is noisy or resembles the foreground exactly. Moreover, the model performs well in JPEG and blurring contexts but still displays minor defects in the finer details. For instance, when distorted, the surfer's forecast is mainly lost under these circumstances, indicating difficulty in segmenting fast‐moving objects in complicated backdrops. Similar hints of some erosion in acceptable accuracy come from minor distortions in items like the man's hand in the fourth row and the horse's leg.

## 5 CONCLUSION

This work presented a new multi‐scale network, CMV2U‐Net, especially for picture splicing forgery detection. Two main constituents of CMV2U‐Net are a splicing forgery localization module and a feature extraction module with double‐stream input. Two separate streams—RGB and edge streams—run through the feature‐extracting module. Using the Canny operator, the edge stream extracts edge information in contrast. These streams improve the localization accuracy of splicing forgers by allowing the model to learn generalized features independent of semantic context. Motivated by a channel attention technique, a hierarchical feature fusion mechanism aggregates the outputs at various layers to enhance the fusing of several streams even more. Edge and contrastive loss, as well as conventional cross‐entropy loss, help to improve feature representation. CMV2U‐Net shows its efficiency and strength through experimental findings on several public datasets, showing that it beats state‐of‐the‐art approaches in splicing forgery localization. In the future, researchers would like to employ advanced hybrid machine learning techniques such as [51, 52, 53, 54] to further improve its performance for bigdatasets in various applications.

## FUNDING INFORMATION

The authors would like to thank their affiliated universities and institutes for supporting this study.

## CONFLICT OF INTEREST STATEMENT

The authors have no conflicts of interest to declare.

## Data Availability

The data will be available upon request to the corresponding author.
